# The miR‐193a‐3p‐MAP3k3 Signaling Axis Regulates Substrate Topography‐Induced Osteogenesis of Bone Marrow Stem Cells

**DOI:** 10.1002/advs.201901412

**Published:** 2019-11-13

**Authors:** Yan Lv, Ying Huang, Mingming Xu, Boon Chin Heng, Congchong Yang, Cen Cao, Zhewen Hu, Wenwen Liu, Xiaopei Chi, Min Gao, Xuehui Zhang, Yan Wei, Xuliang Deng

**Affiliations:** ^1^ Department of Geriatric Dentistry NMPA Key Laboratory for Dental Materials National Engineering Laboratory for Digital and Material Technology of Stomatology Beijing Laboratory of Biomedical Materials Peking University School and Hospital of Stomatology Beijing 100081 P. R. China; ^2^ Department of Cariology and Endodontology Peking University School and Hospital of Stomatology Beijing 100081 P. R. China; ^3^ Department of Stomatology Union Hospital Tongji Medical College Huazhong University of Science and Technology Wuhan 430022 P. R. China; ^4^ Department of Dental Materials and Dental Medical Devices Testing Center Peking University School and Hospital of Stomatology Beijing 100081 P. R. China

**Keywords:** biophysical cues induced‐osteogenic differentiation, miR‐193a‐3p‐MAP3k3 signaling axis, topographical cues

## Abstract

Substrate topographical features induce osteogenic differentiation of bone marrow stem cells (BMSCs), but the underlying mechanisms are unclear. As microRNAs (miRNAs) play key roles in osteogenesis and bone regeneration, it would be meaningful to elucidate the roles of miRNAs in the intracellular signaling cascade of topographical cue‐induced osteogenic differentiation. In this study, the miRNA expression profile of the topographical feature‐induced osteogenic differentiation group is different from that of the chemical‐factors‐induced osteogenic differentiation group. miR‐193a‐3p is sensitive to substrate topographical features and its downregulation enhances osteogenic differentiation only in the absence of osteogenesis−inducing medium. Also, substrate topographical features specifically activate a nonclassical osteogenetic pathway—the mitogen‐activated protein kinase (MAPK) pathway. Loss‐ and gain‐of‐function experiments demonstrate that miR‐193a‐3p regulates the MAPK pathway by targeting the *MAP3k3* gene. In conclusion, this data indicates that different osteogenic‐lineage‐related intracellular signaling cascades are triggered in BMSCs subjected to biophysical or chemical stimulation. Moreover, the miR‐193a‐3p‐MAP3k3 signaling axis plays a pivotal role in the transduction of biophysical cues from the substrate to regulate the osteogenic lineage specification of BMSCs, and hence may be a promising molecular target for bone regenerative therapies.

## Graphical abstract

Topographical feature‐induced bone marrow stem cells (BMSCs) lineage specification is different from that of chemical‐factors‐induced BMSCs lineage specification in terms of microRNA expression. MiR‐193a‐3p‐MAP3k3 signaling axis plays a pivotal role in the transduction of biophysical cues from the substrate to regulate the osteogenic differentiation of BMSCs. As an outlook, when designing implantable biomedical devices or orthopedic substitutes, more attention should be focused on their biophysical properties.

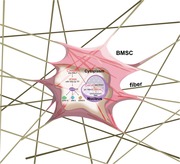

## 1 Introduction

Stem cells reside in a complex microenvironment in which extracellular biophysical cues, such as the topography and stiffness of the extracellular matrix (ECM), as well as various biochemical factors such as growth factors and hormones, play pivotal roles in effecting cell survival, self‐renewal, and differentiation.^1^, ^2^ An understanding of how biophysical cues regulate stem cell function and lineage fate specification would facilitate the design of novel biomaterials to regulate stem cell differentiation and provide guidance for the development of new tissue engineering and regenerative medicine strategies. Stem cells cultured on biomaterials can perceive and respond to topographical features by altering cellular adhesion, cytoskeletal organization, and ultimately cell fate.^3^ However, at present our knowledge of such extracellular signal‐to‐intracellular signal transductions is limited.

microRNAs (miRNAs) are a class of small noncoding RNAs that regulate gene expression at the post‐transcriptional stage by binding to the 3′ untranslated region (UTR) of target mRNAs, which either changes mRNA stability or inhibits protein translation.^4^ miRNAs often simultaneously modulate the activity of several target genes and signaling/regulatory networks, resulting in profound biological effects.^5^ miRNAs are important regulators of gene expression in various organisms and biological/pathological^6^ processes, including the proliferation, migration, and differentiation of bone marrow stem cells (BMSCs). However, the roles of miRNAs in regulating the osteogenesis of BMSCS in response to topographical substrate cues are largely unknown.^7^

In this study, electrospun poly‐l‐lactide (PLLA) membranes with a random nanofibrous arrangement (random group) were utilized to explore the mechanism by which topographical cues in the niche regulate stem cell function and fate. The random arrangement of nanofibers in the scaffold mimics the extracellular collagen distribution and promotes the osteogenic differentiation of human bone marrow‐derived stem cells (hBMSCs). An miRNA chip assay and a bioinformatics/functional analysis were carried out to explore the role of miRNAs in topographical cue‐induced osteogenic differentiation. Substrate stiffness is another important microenvironmental biophysical cue that determines the fate of BMSCs. Therefore, in this study, Gel‐MA hydrogel were utilized as another material model in a preliminary investigation to evaluate whether the miRNAs‐related mechanism elucidated in the PLLA model is of universal significance in biophysical cue‐induced osteogenesis.

## 2 Results

**Figure 1:**
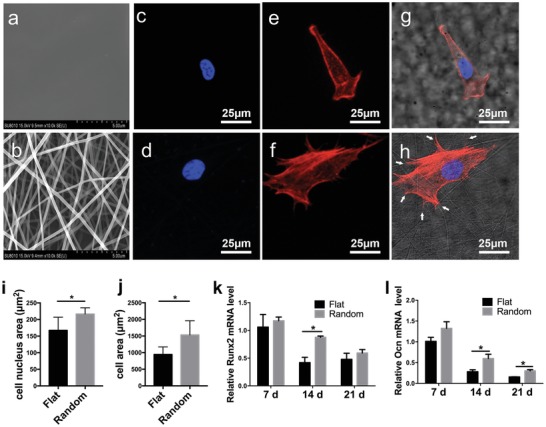
Morphology and osteogenic differentiation of human bone marrow‐derived stem cells (hBMSCs) on poly‐l‐lactide (PLLA) fibrous scaffolds and cast PLLA films. a) Representative scanning electron microscopy images showing the smooth surface in the flat group. b) Representative scanning electron microscopy image of randomly arranged nanofibers (scale bar, 5 µm). c,d) Confocal immunofluorescence staining of nuclei with 4′,6‐diamidino‐2‐phenylindole (DAPI) in hBMSCs from the c) flat group and d) the random fiber group after 4 h in culture. e,f) Confocal immunofluorescence staining of F‐actin with rhodamine‐labeled phalloidin in hBMSCs from the e) flat group and f) the random fiber group after 4 h in culture. g) Merged confocal images of (c) and (e), with bright‐field microscopy images of the flat group. h) Merged confocal microscopy images of (d) and (f), with bright‐field microscopy images of the random group. Scale bar, 25 µm. Quantification of i) the nuclear size and j) cell spreading area of hBMSCs in the flat group and random group (*n* = 30). j) Quantification of hBMSCs in the flat and random groups (*n* = 30). k) Runx2 and l) Ocn mRNA levels in hBMSCs in the flat and random groups at 7, 14, and 21 days, respectively. Results are means ± SEM (*n* = 3). *p < 0.05, * by two‐sample *t*‐test.

Electrospun PLLA nanofibrous substrates promoted the spreading of hBMSCs and enhanced their osteogenic differentiation. We utilized electrospun PLLA membranes with a random fiber arrangement (random group) (**Figure** **1**b) that mimicked the topology of collagen in the ECM as an artificial substrate, to explore the mechanisms by which topographical cues in the niche regulate stem cell function and lineage fate specification. Cast, flat PLLA membranes (flat group) (Figure 1a) were used as the negative control.

The hBMSCs in the random group had cytoplasmic projections that were aligned along individual nanofibers (Figure 1h, white arrows), resulting in an elongated, branched morphology (Figure 1d,f,h). By contrast, the hBMSCs in the flat group displayed a spindle‐like morphology (Figure 1c,e,g). The area of cellular spread and the nuclei were significantly larger in the random compared to the flat group (Figure 1i,j). The expression levels of the osteogenic marker genes *Runx2* and *Ocn* were upregulated in the random group, particularly at 14 days (Figure 1k,l). Therefore, the topological structure of the nanofiber scaffold can induce an osteogenic morphology of hBMSCs, driving their osteogenic differentiation.

**Figure 2:**
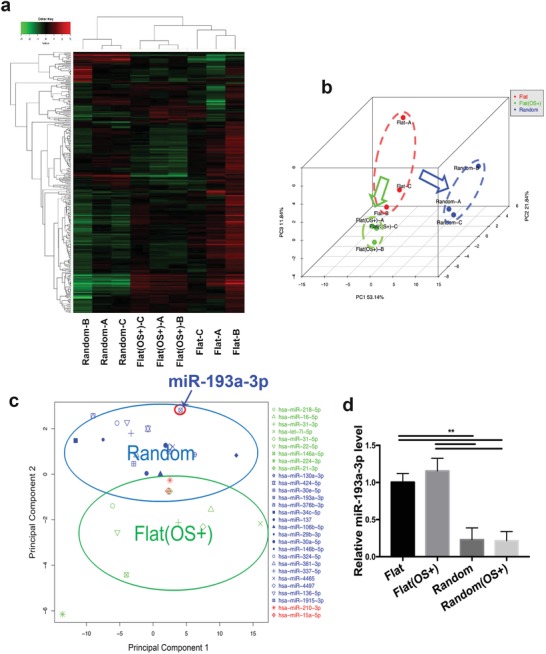
Differential microRNA (miRNA) levels determined using miRNA microarrays and according to component distribution. a) Heatmap of hierarchical clustering and b) 3D PCA of miRNA levels in hBMSCs cultured on the flat, flat (OS+), and random substrates for 14 days. c) The 2D PCA of significantly downregulated miRNAs in the random and flat (OS+) group. d) miR‐193a‐3p levels in hBMSCs from the random, flat (OS+), and random (OS+) groups after 14 days in culture. Results are means ± SEM (*n* = 3). Samples were subjected to one‐way analysis of variance (ANOVA) with Tukey's post hoc test (*p < 0.05, **p < 0.01, ***p < 0.001).

Randomly arranged PLLA fibrous substrates downregulated miR‐193a‐3p expression. Next, miRNA microarrays were used to investigate the role of miRNAs in topographical cue‐induced osteogenic differentiation. hBMSCs in the flat group cultured in chemical osteogenic medium served as the positive control (flat OS+ group). A cluster heatmap analysis showed significantly differentially expressed miRNAs in the random, flat, and flat (OS+) groups (**Figure** **2**a). We next performed a 3D principal component analysis (PCA) to evaluate the spatial distribution of the nine samples from the three groups (Figure 2b). The results showed that hBMSCs in the flat OS+ group and random group have markedly different miRNA profiles.

To identify the miRNAs involved in topographical cue‐activated osteogenic differentiation, we screened out 9 significantly downregulated miRNAs in the flat OS+ group, together with 19 downregulated miRNAs in the random group (2.5‐fold or greater difference in expression level compared to the flat group). In the 2D PCA of significantly downregulated miRNAs, the component distribution that represented the characteristic downregulated miRNAs in the random group (blue circle) differed from that of the flat (OS+) group (green circle) (Figure 2c). This suggests that topographically activated osteogenic differentiation differs from that induced biochemically. The 2D PCA showed that miR‐193a‐3p was significantly downregulated in the random group, which is the farthest from the center of the flat (OS+) group. Thus, we selected miR‐193a‐3p as a representative miRNA for further analysis. Next, quantitative polymerase chain reaction (qPCR) showed that miR‐193a‐3p expression was downregulated in the random group irrespective of use of chemical osteogenic medium, while there was no significant difference between the flat (OS+) group and flat group after 14 days in culture (Figure 2d). These results indicate that miR‐193a‐3p is downregulated by topological activation and is involved in the cellular sensing of changes in surface topography.

**Figure 3:**
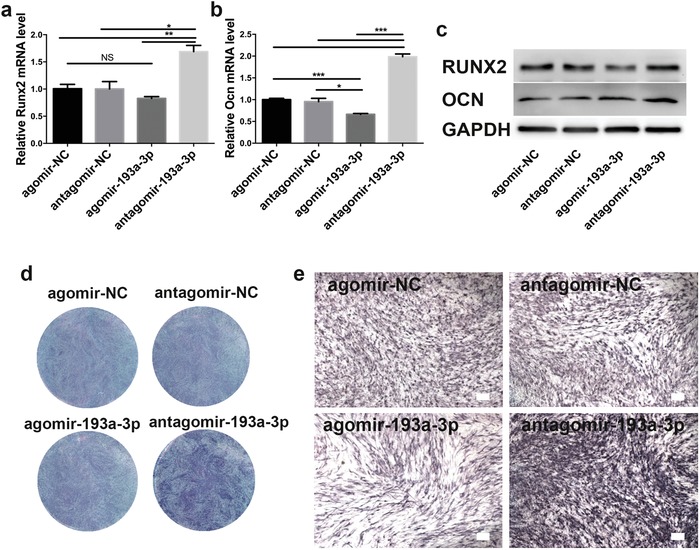
Downregulation of miR‐193a‐3p correlates with enhancement of the osteogenic differentiation of hBMSCs. a–c) Effects of agomir‐193a‐3p, antagomir‐193a‐3p, and their scrambled controls on the mRNA levels of a) *Runx2* and b) *Ocn*, and their protein levels c) in hBMSCs. d) Gross and e) magnified images of alkaline phosphatase (ALP) staining of hBMSCs treated with agomir‐193a‐3p, antagomir, or their scrambled controls for 5 days. Results are means ± SEM (*n* = 3). Samples were subjected to one‐way ANOVA with Tukey's post hoc test (*p < 0.05, **p < 0.01, ***p < 0.001).

Downregulation of miR‐193a‐3p promoted the osteogenic differentiation of hBMSCs. Next, we investigated the role of miR‐193a‐3p in the function of hBMSCs by treating them with agomir‐193a‐3p (a miR‐193a‐3p agonist) and antagomir‐193a‐3p (a miR‐193a‐3p inhibitor), together with their scrambled controls. Intracellular miR‐193a‐3p was markedly upregulated by agomir‐193a‐3p, and markedly downregulated by antagomir‐193a‐3p (Figure S1, Supporting Information). qPCR analysis showed that the *Runx2* and *Ocn* mRNA levels were markedly upregulated by antagomir‐193a‐3p, but downregulated by agomir‐193a‐3p, when compared to the corresponding scrambled controls (**Figure** **3**a,b). Functionally, antagomir increased the protein expression levels of RUNX2 and OCN, while agomir decreased the protein expression levels of RUNX2 (Figure 3c; Figure S2, Supporting Information). Consistently, overexpression of miR‐193a‐3p weakened ALP staining, while knockdown of miR‐193a‐3p enhanced it (Figure 3e,f). Interestingly, agomir‐193a‐3p did not decrease the protein expression levels of OCN (Figure 3c; Figure S2b, Supporting Information). These results thus revealed that downregulation of miR‐193a‐3p expression promotes the osteogenic differentiation of hBMSCs.

**Figure 4:**
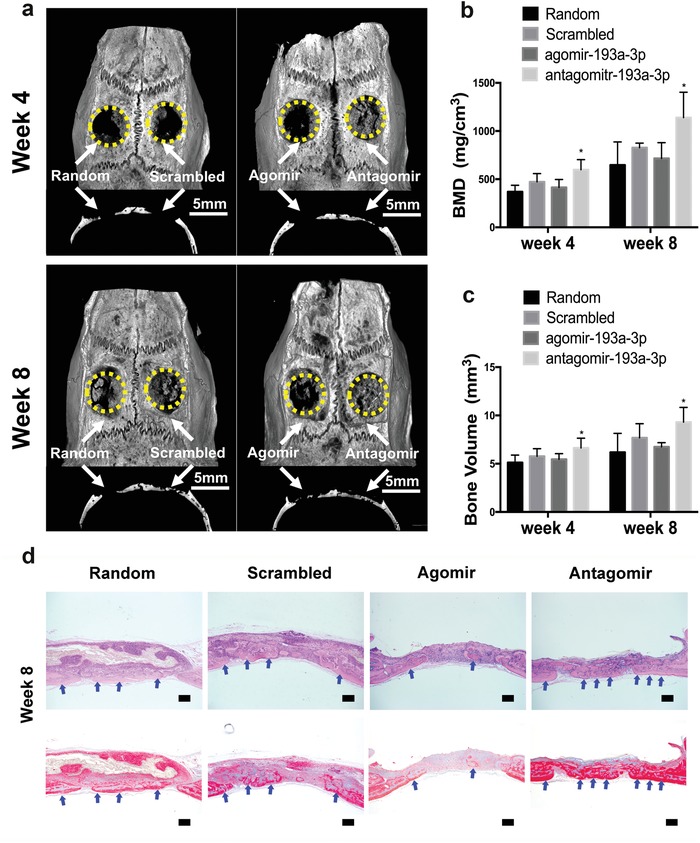
Nanofiber membranes loaded with miR‐193a‐3p antagomir enhanced the healing of critical‐sized bone defects. a) Representative micro‐CT images and sagittal views of rat cranial critical‐sized full‐thickness defects at 4 and 8 weeks after surgery (scale bar, 5 mm). Yellow circles and white arrows indicate the bone defect area. b,c) Quantitative analysis of BV and BMD of the newly formed bone. Data are means ± SE) (*n* = 6) and all *p*‐values are based on one‐way ANOVA with a post hoc test (*p < 0.05). d) Histological results of 8‐weeks H&E staining (Top row) and Masson staining (Bottom row). Blue arrows denote the newly‐formed bone. (bar = 200 µm)

miR‐193a‐3p antagomir‐loaded nanofiber scaffolds enhanced the healing of critical‐sized bone defects in rat cranium. To confirm the role of miR‐193a‐3p in bone defect healing, we covered critical‐sized bone defects in rat cranium with four membranes composed of PLLA nanofiber scaffolds loaded by lyophilization with i) agomir‐193a‐3p, ii) antagomir‐193a‐3p, iii) scrambled control, and iv) water. After 4 weeks, microcomputed tomography (micro‐CT) image reconstruction showed that in the group with PLLA membranes loaded with antagomir‐193a‐3p, newly formed high‐density bone almost filled the defect, while there was scant newly formed bone in the groups loaded with agomir‐193a‐3p, scrambled control, and blank (**Figure** **4**a). At 8 weeks after implantation, the bone defect in the antagomir‐193a‐3p‐loaded group had almost completely healed, while markedly less newly formed low‐density bone was found in the groups loaded with scrambled control and blank. Only minimal new bone formation occurred in the group loaded with agomir‐193a‐3p (Figure 4a). Quantitative analysis showed that both the regenerated bone volume (BV) and the bone mineral density (BMD) were highest in the antagomir‐193a‐3p group (Figure 4b,c). The results of the histological analysis corroborate the micro‐CT data (Figure 4d). At 8 weeks postimplantation, the bone defect in the antagomir‐193a‐3p‐loaded group had almost completely healed with obvious bone‐structure formation, while less newly‐formed bone was observed in the groups loaded with scrambled control and blank. Only minimal new bone formation was observed in the group loaded with agomir‐193a‐3p, with obvious unrepaired defect areas. Therefore, these results showed that downregulation of miR‐193a‐3p markedly enhanced the healing of critical‐sized bone defects.

**Figure 5:**
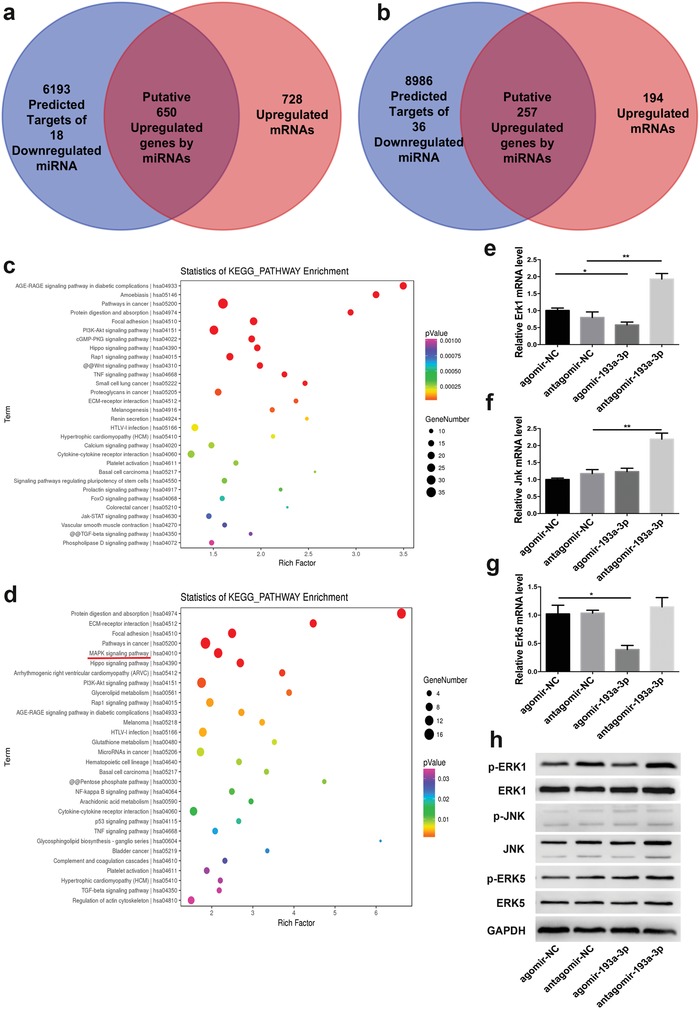
PLLA nanofibrous substrates activated the mitogen‐activated protein kinase (MAPK) signaling pathway by downregulating miR‐193a‐3p. a,b) miRNA–mRNA integrated analysis of the flat (OS+) (a) random groups (b). c) Significantly enriched pathways for the 650 putatively upregulated genes in (a) (*p* < 0.001). d) Significantly enriched pathways for the 257 putatively upregulated genes in (b) (*p* < 0.05). e–g) mRNA and h) protein levels of MAPK kinases in bone marrow stem cells (BMSCs) treated with agomir, antagomir, or their scrambled controls. Results are means ± SEM (*n* = 3). Samples were subjected to one‐way ANOVA with Tukey's post hoc test (*p < 0.05, **p < 0.01).

miR‐193a‐3p regulates the mitogen‐activated protein kinase (MAPK) signaling pathway. The miRNA–mRNA integrated assay was used to analyze the pathways regulated by miRNAs during the osteogenic differentiation of BMSCs activated by topological cues. Eighteen miRNAs were more than twofold significantly downregulated in BMSCs in the flat (OS+) group versus the flat group, and thirty‐six miRNAs were significantly downregulated more than twofold in the random group versus the flat group. We next compared the predicted 6843 target genes of the 18 downregulated miRNAs in the flat (OS+) group with the 1378 upregulated (≥1.5‐fold) genes identified by microarray analysis in our previous study.^8^ We found that 650 upregulated genes (**Figure** **5**a) overlapped. We also compared the predicted 9243 target genes of the 36 downregulated miRNAs in the random group with the 451 upregulated (≥1.5‐fold) genes identified by microarray analysis in our previous study.^8^ We found that 257 upregulated genes overlapped (Figure 5b). To explore the intracellular signaling pathways to which these genes belong, we performed an ontology analysis using KOBAS 3.0 software (http://kobas.cbi.pku.edu.cn/anno_iden.php) (Figure 5c,d). A pathway enrichment analysis revealed substantial differences between the two groups. Comparison of the top 10 pathways between the two groups (Tables S1 and S2, Supporting Information) revealed that 6 overlapped (Figure S3a, Supporting Information). Among the top 20 pathways in the two groups (Tables S1 and S2, Supporting Information), only 9 overlapped (Figure S3b, Supporting Information) and 14 were found to be consistently enriched (Figure S3c, Supporting Information). Overall, there was only 50% similarity between the two groups, indicating marked differences between the physical and chemical activation of osteogenesis. A gene enrichment analysis showed that the MAPK pathway was the top pathway in the random group but not in the flat (OS+) group. Hence, MAPK signaling is likely involved in activation of the osteogenic differentiation of BMSCs by topographical cues. As stated above, miR‐193a‐3p was involved in topological cue‐activated osteogenic differentiation of BMSCs, and we hypothesized that activation of the MAPK signaling pathway and miR‐193a‐3p expression are closely correlated. Interestingly, some key factors in the MAPK pathway, such as those encoded by *Erk1* and *Jnk*, are predicted by bioinformatics to be target genes of miR‐193a‐3p. We found that antagomir‐193a‐3p significantly increased the mRNA levels of *Erk1* and *Jnk*, while agomir‐193a‐3p significantly decreased the mRNA levels of *Erk1* and *Erk5* (Figure 5e–g). The protein expression levels of ERK1, JNK, p‐ERK1, and p‐ERK5 were increased by antagomir, while the expression levels of ERK1, JNK, and p‐ERK1 were decreased by agomir‐193a‐3p (Figure 5h). These results verified that miR‐193a‐3p suppresses the MAPK pathway.

**Figure 6:**
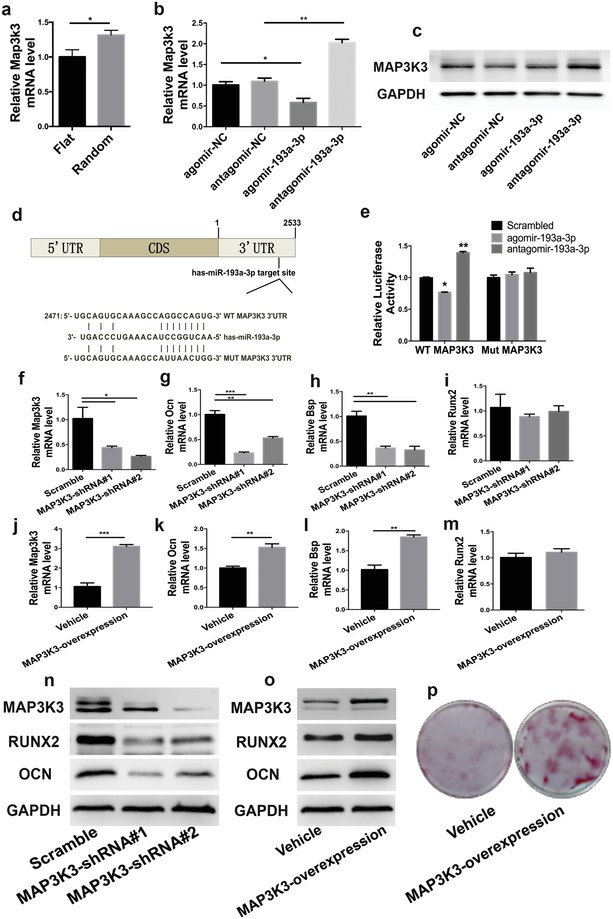
miR‐193a‐3p directly targeted MAP3K3. a) Map3k3 mRNA expression levels of hBMSCs in the flat and random groups. b) mRNA and c) protein levels of MAP3K3 in hBMSCs treated with agomir‐193a‐3p, antagomir‐193a‐3p, and their corresponding scrambled controls. d) Schematic diagram of the design of luciferase reporters with the wild‐type Map3k3 3′UTR (WT MAP3K3 3′UTR) or the site‐directed mutant Map3k3 3′UTR (MUT MAP3K3 3′UTR). e) The effects of agomir‐193a‐3p, antagomir‐193a‐3p, or their corresponding scrambled controls on the luciferase activity of WT MAP3K3 3′UTR and MUT MAP3K3 3′UTR reporter in hBMSCs. f) Transfection efficiency of Map3k3‐small‐hairpin RNA (shRNA). g–i) mRNA levels of osteogenic genes in hBMSCs treated with MAP3K3‐shRNA. j) Transfection efficiency of the Map3k3‐overexpression vector. k–m) mRNA levels of osteogenic genes in MAP3K3 overexpressing hBMSCs. n). Protein levels of osteogenic factors in hBMSCs treated with MAP3K3‐shRNA. o) Protein levels of osteogenic factors in MAP3K3 overexpressing hBMSCs. p) Alizarin red staining of MAP3K3 overexpressing hBMSCs after 21 days of culture. Data are means ± SD (*n* = 3). *p < 0.05, **p < 0.01, ***p < 0.001.

miR‐193a‐3p directly targeted MAP3K3. To gain insight into the mechanisms by which miR‐193a‐3p regulates the MAPK pathway activity and affects the osteogenic differentiation of BMSCs, we used miRWalk 2.0 (http://zmf.umm.uni-heidelberg.de/apps/zmf/mirwalk2/index.html) to predict its potential targets. Among the candidate target genes, Map3k3 has a miR‐193a‐3p–binding site at its 3′ UTR. Next, we verified that the Map3k3 mRNA level in hBMSCs was upregulated in the random group (**Figure** **6**a). We found that the *Map3k3* mRNA level (assessed by qPCR) was downregulated by agomir‐193a‐3p but upregulated by antagomir‐193a‐3p, as compared to the corresponding scrambled controls (Figure 6b). Also, agomir decreased, and antagomir increased, the MAP3K3 protein level (Figure 6c).

To further confirm the miR‐193a‐3p target region in the *Map3k3* mRNA, we constructed MAP3K3 3′UTR luciferase reporters that contained wild‐type (WT MAP3K3 3′UTR reporter) and mutant (MUT MAP3K3 3′UTR reporter) sequences of the miR‐193a‐3p binding sites (Figure 6d), and co‐transfected hBMSCs with these together with miR‐193a‐3p oligonucleotides. We found that agomir‐193a‐3p decreased, while antagomir‐193a‐3p increased, the luciferase reporter activity of WT MAP3K3 3′UTR, but not that of MUT MAP3K3 3′UTR (Figure 6e). Therefore, miR‐193a‐3p directly targets Map3k3 and binds to its 3′UTR.

Next, we knocked down Map3k3 in hBMSCs using Map3k3‐targeting small‐hairpin RNAs (shRNAs) to disrupt the expression of the Map3k3 gene (Figure 6f), and used a lentivirus to overexpress the Map3k3 gene (Figure 6j). We found that the mRNA levels of *Ocn* and *Bsp* were significantly decreased by *Map3k3* knockdown (Figure 6g,h) and that the expression levels of these genes were markedly increased by *Map3k3* overexpression (Figure 6k,l). But the mRNA levels of *Runx2* remained unchanged with either *Map3k3* knockdown (Figure 6i) or overexpression (Figure 6m). The protein levels of OCN and RUNX2 were significantly decreased by MAP3K3 knockdown but markedly increased by its overexpression (Figure 6n,o). Increased Alizarin red staining in the MAP3K3 overexpression group was observed after 21 days in the absence of chemical osteogenic medium (Figure 6p). Therefore, miR‐193a‐3p regulated topographical feature‐activated osteogenic differentiation of hBMSCs in the random group by directly binding to *Map3k3*.

**Figure 7:**
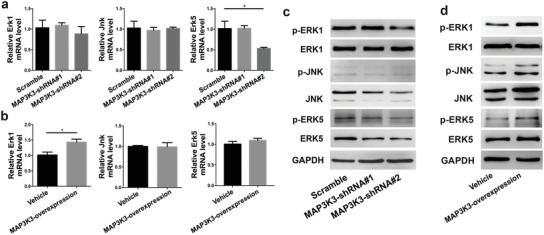
MAP3K3 activates the MAPK signaling pathway. a) mRNA levels of MAPK kinases upon MAP3K3 downregulation by lentivirus‐shRNA. b) mRNA levels of MAPK kinases upon MAP3K3 overexpression by lentivirus‐MAP3K3. c) Protein levels of MAPK kinases upon MAP3K3 downregulation by lentivirus‐shRNA. d) Protein levels of MAPK kinases upon MAP3K3 overexpression by lentivirus‐MAP3K3. Data are means ± SD (*n* = 3). All *p*‐values by Student's *t*‐test. *p < 0.05.

miR‐193a‐3p activated the MAPK signaling pathway through the miR‐193a‐3p‐MAP3K3 axis. We have shown that miR‐193a‐3p was downregulated in the random group (Figure 2), while the miRNA–mRNA integrated analysis indicated that the MAPK signaling pathway was enriched in the random group and that miR‐193a‐3p expression was negatively correlated with activation of the MAPK signaling pathway (Figure 5). *Map3K3* is a target gene of miR‐193a‐3p (Figure 6). Next, we investigated the effect of loss‐ and gain‐of‐function of MAP3K3 on the MAPK pathway. We used a lentivirus to knock down and overexpress MAP3K3 in hBMSCs. The qPCR results showed that the mRNA level of only *Erk5* was significantly decreased after *Map3K3* knockdown (**Figure** **7**a). Also, the mRNA level of only Erk1 was significantly increased by *Map3K3* overexpression (Figure 7b). The results of Western blotting showed that the JNK, ERK5, p‐ERK1, p‐JNK, and p‐ERK5 levels were markedly decreased by MAP3K3 knockdown (Figure 7c). Conversely, the levels of JNK, ERK5, p‐ERK1, p‐JNK, and p‐ERK5 were significantly increased by MAP3K3 overexpression (Figure 7d). The protein level of ERK1 was unaffected by knock down or overexpression of MAP3K3 in hBMSCs (Figure 7c,d). These results suggest that miR‐193a‐3p negatively regulates, and MAP3K3 positively regulates, the MAPK pathway, and that both regulate the activation (or phosphorylation) of ERK1, JNK, and ERK5, which are key signaling molecules in the MAPK pathway. Next, we co‐transfected MAP3K3‐shRNA lentivirus and agomir‐193a‐3p or antagomir‐193a‐3p into hBMSCs to verify the relationships of miR‐193a‐3p, Map3k3, and Ocn. The qPCR results showed that antagomir‐193a‐3p upregulated the *Map3k3* and *Ocn* mRNA levels, and that MAP3K3‐shRNA interfered with this effect (Figure S4, Supporting Information). Therefore, miR‐193a‐3p activates the MAPK pathway through the miR‐193a‐3p‐MAP3K3 signaling axis.

**Figure 8:**
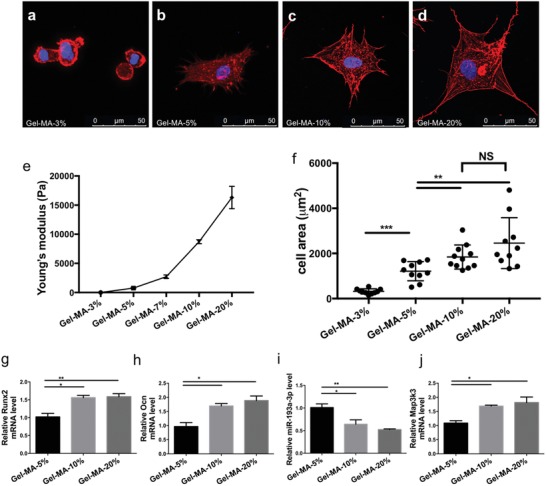
miR‐193a‐3p mediated hBMSC osteogenic differentiation in response to substrate stiffness on gelatin methacrylate (Gel‐MA). a–d) Confocal immunofluorescence of cytoskeletal actin and nuclear DNA of hBMSCs cultured on Gel‐MA with different substrate stiffnesses at 24 h postseeding (scale bar, 50 µm). Actin, red; nuclear DNA, blue. e) The stiffness of the Gel‐MA hydrogel could be modified by altering its concentration; the higher the gel concentration, the higher the Young's modulus. f) Cell spreading areas on substrates with different stiffnesses (*n* = 10). g–j) mRNA levels of *Runx2*, *Ocn*, miR‐193a‐3p, and *Map3k3* in cells cultured on substrates with different stiffnesses (*n* = 3). Results are means ± SEM. Samples were subjected to one‐way ANOVA with Tukey's post hoc test. Significant differences are denoted by *p < 0.05.

The miR‐193a‐3p‐MAP3K3 axis modulates the osteogenic differentiation of hBMSCs in response to substrate rigidity. Substrate stiffness is a microenvironmental biophysical cue that determines the fate of BMSCs and has been the focus of recent research.^9^ In this study, Gel‐MA hydrogel^10^ was utilized to investigate the role of miR‐193a‐3p in substrate stiffness‐induced osteogenic differentiation of hBMSCs. We found that the numbers of pseudopod protrusions and the activation of cytoskeletal components of hBMSCs increased as matrix stiffness increased. hBMSCs displayed a globular‐like phenotype on the 3% (w/v) Gel‐MA hydrogel with a poorly developed cytoskeleton, while hBMSCs cultured on the 20% (w/v) Gel‐MA hydrogel were polygonal and had an osteoblast‐like morphology with a well‐developed cytoskeleton (**Figure** **8**a–d). Also, the spread of the hBMSCs increased with increasing matrix stiffness (Figure 8e,f). The mRNA levels of the osteogenic marker genes *Runx2* and *Ocn* also increased with increasing matrix stiffness (Figure 8g,h). These phenotypes indicated that biophysical cues, such as matrix stiffness, determine the fate of BMSCs by altering their adhesion morphology. Next, we investigated the role of miR‐193a‐3p and its target gene *Map3k3* in the substrate stiffness‐induced osteogenic differentiation of hBMSCs. We found that miR‐193a‐3p was downregulated, but its target gene *Map3k3* was upregulated, in the groups with higher Gel‐MA stiffness (Figure 8i,j). Therefore, the miR‐193a‐3p‐MAP3K3 signaling axis may be involved in the substrate stiffness‐induced osteogenic differentiation of BMSCs.

## 3 Discussion

Biomaterials can be tailored to provide a microenvironment that directs the differentiation of stem cells into the desired lineages.^1^, ^11^ The physical properties of biomaterials can also modulate stem cell differentiation and lineage fate by means of cellular mechanosensing and/or other mechanisms.^12^ The data presented in this study revealed an miRNA‐related mechanism for topographical feature‐induced osteogenic differentiation of BMSCs. We found that downregulation of miR‐193a‐3p expression was specifically involved in substrate topography‐induced osteogenic differentiation of hBMSCs. miR‐193a‐3p can directly bind to *Map3k3* mRNA, which results in downregulation of *Map3k3* expression. On substrates with topographical features, miR‐193a‐3p expression was downregulated, leading to upregulation of *Map3k3* and subsequent activation of the MAPK signaling pathway, which promoted the osteogenic differentiation of hBMSCs. We further found that the miR‐193a‐3p‐MAP3K3 signaling axis plays a role in modulating substrate‐rigidity‐induced osteogenic differentiation. Hence, the miR‐193a‐3p‐MAP3K3 signaling axis is needed for transduction of biophysical stimuli from the substrate to induce the osteogenic differentiation of hBMSCs.

Osteogenic differentiation trigged by biophysical and chemical cues are different processes. During biophysical cue‐stimulated osteogenic differentiation, a change in focal adhesion behavior and cytoskeleton re‐arrangement occur before any alteration of gene expression,^13^, ^14^ while in chemical cue‐induced osteogenic differentiation, gene expression changes before cytoskeleton re‐arrangement and a shift in cell morphology.^15^ The different miRNA expression profiles of the random and flat (OS+) groups further suggest that biophysical and chemical induction of osteogenic differentiation are mechanistically different (Figure 2a,b). We found that some miRNAs are sensitive to biophysical, but insensitive to chemical osteogenic induction (Figure 2c,d). Also, the topographical features in the random group downregulated the pri‐ and pre‐miRNA levels, suggesting that the different miRNA expression profile in the random group is due to the effect of topographical features on the biogenesis of miRNA (Figure S5a,b, Supporting Information).

We found that the miR‐193a‐3p‐MAP3K3‐MAPK axis mediated topographical feature‐induced osteogenic differentiation. This axis also plays a role in the osteogenesis induced by other biophysical cues, as similar results were obtained using a second biophysical material (Figure 8). BMP/Smad,^16^, ^17^ Wnt,^18^ and TGF‐β^16^, ^19^ are the classical signaling pathways involved in chemical‐induced osteogenic differentiation, and drive the osteogenic differentiation of stem cells.^16^, ^17^ The MAPK signaling pathway is activated by various extracellular stimuli, including radiation, osmotic pressure, temperature, and mechanical force, and regulates cell proliferation, differentiation, and survival.^20^ It is not a typical signaling pathway driving the osteogenic differentiation of stem cells, in agreement with the finding that biophysical cues induce only moderate osteogenic differentiation.^21^ In this study, we found that the role of miR‐193a‐3p in chemical‐induced osteogenic differentiation is likely obscured (Figure S6, Supporting Information). Thus, the results of this study deepen our understanding of how biophysical cues determine the lineage into which stem cells differentiate.

In this study, the downregulation of miR‐193a‐3p enhanced the healing of the critical‐sized bone defects (Figure 4). This data showed that miR‐193a‐3p could be used as a molecular switch to regulate mechanosensing of biophysical cues in vivo. Treatment with miR‐193a‐3p antagomir mimicked the mechanosensing of biophysical cues such as topographical features and matrix stiffness. As a result, the healing of critical‐sized bone defect was enhanced. This phenomenon also suggested that when designing implantable biomedical devices or orthopedic substitutes, more attention should be focused on their biophysical properties, including their topographical features and elastic modulus.

In summary, the miRNA expression profiles differed between biophysical‐ and chemical‐induced differentiation. Also, some miRNAs were sensitive to biophysical, but insensitive to chemical, induction of osteogenesis. We found that the miR‐193a‐3p‐MAP3K3‐MAPK axis mediated topographical feature‐induced osteogenic differentiation, and that this signaling axis is common to osteogenesis induced by other biophysical cues. The results of this study deepen our understanding of how biophysical cues determine the lineage into which stem cells will differentiate.

## Conflict of Interest

The authors declare no conflict of interest.

## Author Contributions

Y.L. and Y.H. contributed equally to this work. Y.L., Y.H., X.Z., Y.W., and X.D. conceived the idea for the study. Y.L., Y.H., M.X., C.Y., and C.C. collected the data. Y.L., Y.H., Z.H., X.C., M.G., and W.L. discussed, and contributed to, the final design of the study. Y.L. and Y.H. wrote the first draft of the manuscript with significant assistance from B.C.H. and X.D. All the authors contributed to the completion and revision of the manuscript.

## Supporting information

Supporting Information

## References

[1] M. P. Lutolf , P. M. Gilbert , H. M. Blau , Nature 2009, 462, 433. 19940913 10.1038/nature08602 PMC2908011

[2] a) D. E. Discher , D. J. Mooney , P. W. Zandstra , Science 2009, 324, 1673; 19556500 10.1126/science.1171643 PMC2847855

[3] a) J. Fiedler , B. Ozdemir , J. Bartholoma , A. Plettl , R. E. Brenner , P. Ziemann , Biomaterials 2013, 34, 8851; 23968851 10.1016/j.biomaterials.2013.08.010

[4] a) V. Ambros , Nature 2004, 431, 350; 15372042 10.1038/nature02871

[5] a) D. P. Bartel , Cell 2009, 136, 215; 19167326 10.1016/j.cell.2009.01.002 PMC3794896

[6] H. Song , X. Li , Z. Zhao , J. Qian , Y. Wang , J. Cui , W. Weng , L. Cao , X. Chen , Y. Hu , J. Su , Nano Lett. 2019, 19, 3040. 30968694 10.1021/acs.nanolett.9b00287

[7] J. L. Yang , L. E. McNamara , N. Gadegaard , E. V. Alakpa , K. V. Burgess , R. M. D. Meek , M. J. Dalby , ACS Nano 2014, 8, 9941. 25227207 10.1021/nn504767g

[8] W. T. Liu , Y. Wei , X. H. Zhang , M. M. Xu , X. P. Yang , X. L. Deng , ACS Nano 2013, 7, 6928. 23906375 10.1021/nn402118s

[9] K. H. Vining , D. J. Mooney , Nat. Rev. Mol. Cell Biol. 2017, 18, 728. 29115301 10.1038/nrm.2017.108 PMC5803560

[10] a) M. Rizwan , G. S. L. Peh , H.‐P. Ang , N. C. Lwin , K. Adnan , J. S. Mehta , W. S. Tan , E. K. F. Yim , Biomaterials 2017, 120, 139; 28061402 10.1016/j.biomaterials.2016.12.026

[11] L. Wan , H. Song , X. Chen , Y. Zhang , Q. Yue , P. Pan , J. Su , A. A. Elzatahry , Y. Deng , Adv. Mater. 2018, 30, 1707515. 10.1002/adma.201707515 29733478

[12] a) A. Mammoto , T. Mammoto , D. E. Ingber , J. Cell Sci. 2012, 125, 3061; 22797927 10.1242/jcs.093005 PMC3434847

[13] G. Abagnale , A. Sechi , M. Steger , Q. H. Zhou , C. C. Kuo , G. Aydin , C. Schalla , G. Muller‐Newen , M. Zenke , I. G. Costa , P. van Rijn , A. Gillner , W. Wagner , Stem Cell Rep. 2017, 9, 654. 10.1016/j.stemcr.2017.06.016 PMC5550028 28757164

[14] a) E. K. F. Yim , E. M. Darling , K. Kulangara , F. Guilak , K. W. Leong , Biomaterials 2010, 31, 1299; 19879643 10.1016/j.biomaterials.2009.10.037 PMC2813896

[15] a) M. D. Treiser , E. H. Yang , S. Gordonov , D. M. Cohen , I. P. Androulakis , J. Kohn , C. S. Chen , P. V. Moghe , Proc. Natl. Acad. Sci. USA 2010, 107, 610; 20080726 10.1073/pnas.0909597107 PMC2818905

[16] M. R. Wu , G. Q. Chen , Y. P. Li , Bone Res., https://doi.org/1038/boneres.2016.9.

[17] J. W. Lowery , V. Rosen , Physiol. Rev. 2018, 98, 2431. 30156494 10.1152/physrev.00028.2017

[18] a) R. Baron , M. Kneissel , Nat. Med. 2013, 19, 179; 23389618 10.1038/nm.3074

[19] a) Y. Tang , X. W. Wu , W. Q. Lei , L. J. Pang , C. Wan , Z. Q. Shi , L. Zhao , T. R. Nagy , X. Y. Peng , J. B. Hu , X. Feng , W. Van Hul , M. Wan , X. Cao , Nat. Med. 2009, 15, 757; 19584867 10.1038/nm.1979 PMC2727637

[20] L. Hoffman , C. C. Jensen , M. Yoshigi , M. Beckerle , Mol. Biol. Cell 2017, 28, 2661. 28768826 10.1091/mbc.E17-02-0087 PMC5620374

[21] A. Schindeler , D. G. Little , J. Bone Miner. Res. 2006, 21, 1331. 16939391 10.1359/jbmr.060603

