# Supplementary material for: The miR‐193a‐3p‐MAP3k3 Signaling Axis Regulates Substrate Topography‐Induced Osteogenesis of Bone Marrow Stem Cells
Source: Adv Sci (Weinh). 2019 Nov 13;7(1):1901412. doi: 10.1002/advs.201901412 (PMC6947707; doi:10.1002/advs.201901412)
Supplement: Supplementary file 1 — Supporting Information [file ADVS-7-1901412-s001.pdf]

Copyright WILEY-VCH Verlag GmbH & Co. KGaA, 69469 Weinheim, Germany, 2019.

ADVANCED SCIENCE
Open Access

Supporting Information

for *Adv. Sci.*, DOI: 10.1002/advs.201901412

The miR-193a-3p-MAP3k3 Signaling Axis Regulates Substrate Topography-Induced Osteogenesis of Bone Marrow Stem Cells

*Yan Lv, Ying Huang, Mingming Xu, Boon Chin Heng, Congchong Yang, Cen Cao, Zhewen Hu, Wenwen Liu, Xiaopei Chi, Min Gao, Xuehui Zhang, Yan Wei, and Xuliang Deng**

Supplementary information

The miR-193a-3p-MAP3k3 signaling axis regulates substrate topography-induced osteogenesis of bone marrow stem cells

*Yan Lv, Ying Huang, Mingming Xu, Boon Chin Heng, Congchong Yang, Cen Cao, Zhewen Hu, Wenwen Liu, Xiaopei Chi, Min Gao, Xuehui Zhang, Yan Wei, and Xuliang Deng**

Dr. Y. Lv, Dr. Y. Huang, Dr. M. Xu, Prof. B. C. Heng, Dr. Z. Hu, Dr. X. Chi, Dr. M. Gao, Dr. W. Liu, Prof. Y. Wei and Prof. X. Deng

Department of Geriatric Dentistry, NMPA Key Laboratory for Dental Materials, National Engineering Laboratory for Digital and Material Technology of Stomatology, Beijing Laboratory of Biomedical Materials, Peking University School and Hospital of Stomatology, Beijing 100081, PR China.

Dr. C. Yang

Department of Cariology and Endodontology, Peking University School and Hospital of Stomatology, Beijing 100081, P. R. China.

Dr. C. Cao

Department of Stomatology, Union Hospital, Tongji Medical College, Huazhong University of Science and Technology, Wuhan 430022, China.

Dr. X. Zhang

Department of Dental Materials & Dental Medical Devices Testing Center, Peking

University School and Hospital of Stomatology, Beijing 100081, P. R. China.

Dr. Y. Lv and Dr. Y. Huang contributed equally to this paper.

*Corresponding author: Prof. Xuliang Deng, Department of Geriatric Dentistry, Peking University School and Hospital of Stomatology, 22# Zhongguancun South Ave, Haidian District, Beijing, 100081, P.R. China

Phone: 86 10-82195637

Fax: 86 10-82195581

Email: kqdengxuliang@bjmu.edu.cn

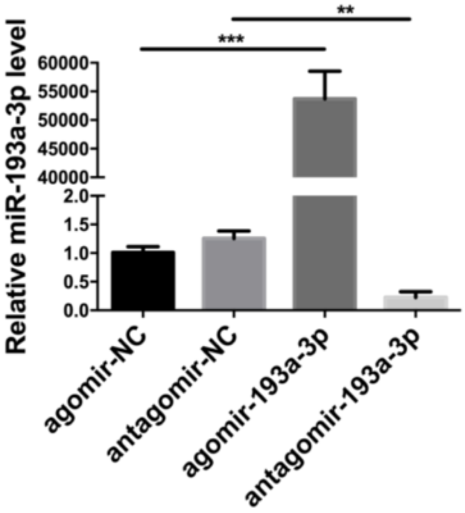

Supplementary Fig. 1. The expression of miR-193a-3p at 48 hours post-transfection in basal medium. Results are presented as means ± SEM (n = 3). $^{**}p < 0.01$, $^{***}p < 0.001$, * by two-sample *t*-test.

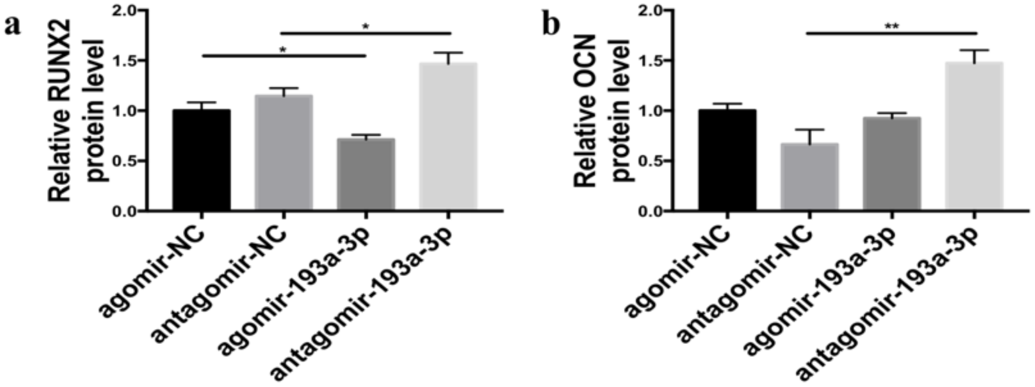

Supplementary Fig. 2. Quantitative analysis of the protein expression levels of RUNX2 (a) and OCN (b). Results are presented as means ± SEM (n = 3). *$p < 0.05$, **$p < 0.01$, * by two-sample t-test.

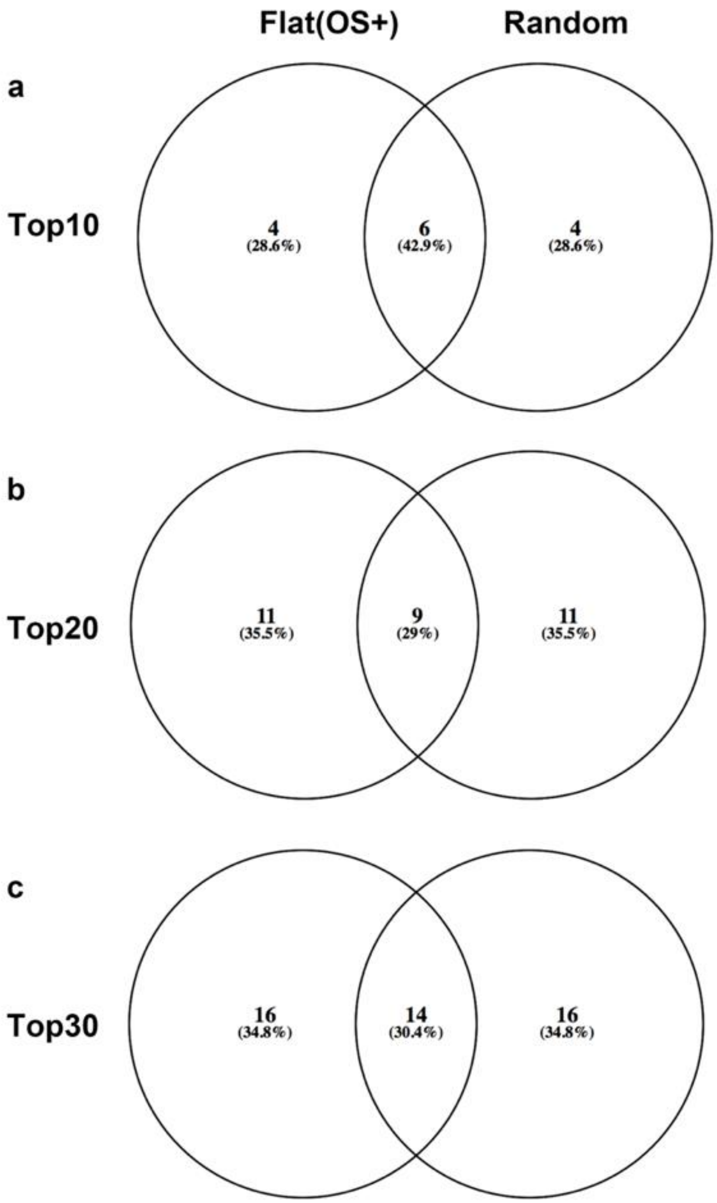

Supplementary Fig. 3. Enrichment pathways in the flat (OS+) and random groups. (**a**) Top 10 enriched pathways in the flat (OS+) and random groups. (**b**) Top 20 enriched pathways in the flat (OS+) and random groups. (**c**) Top 30 enriched pathways in the flat (OS+) and random groups.

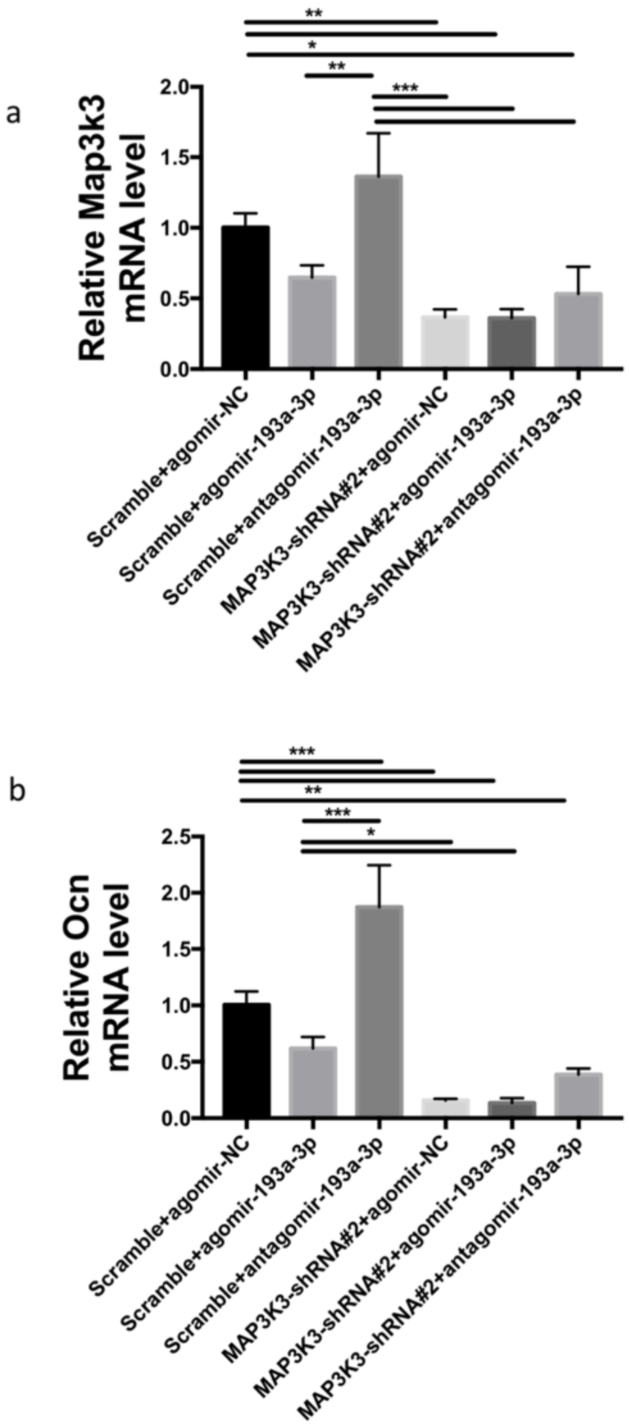

Supplementary Fig. 4. MAP3K3 was downregulated by co-transfection of lentivirus-shRNA with agomir-193a-3p or antagomir-193a-3p. (**a**) mRNA level of *Map3k3* following co-transfection of lentivirus-shRNA with agomir-193a-3p or antagomir-193a-3p. (**b**) mRNA level of *Ocn* upon co-transfection of lentivirus-shRNA with agomir-193a-3p or antagomir-193a-3p. Results are presented as means ± SEM (n = 3). Samples were subjected to one-way ANOVA with Tukey's *post hoc* test. Significant differences are denoted by *$p < 0.05$, **$p < 0.01$, and ***$p < 0.001$.

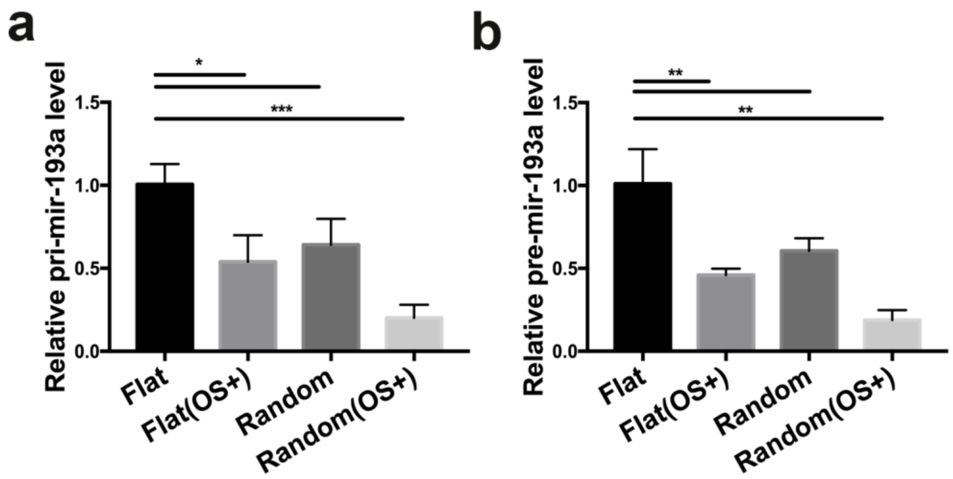

Supplementary Fig. 5. Expression levels of mir-193a and miR-193a-3p. Pri-mir-193a (**a**) and pre-mir-193a (**b**) expression in hBMSCs in the flat (OS+), random, and random (OS+) groups at 14 days. Results are means ± SEM (n = 3). Samples were subjected to one-way ANOVA with Tukey's *post hoc* test. Significant differences are denoted by $*p < 0.05$, $**p < 0.01$, and $***p < 0.001$.

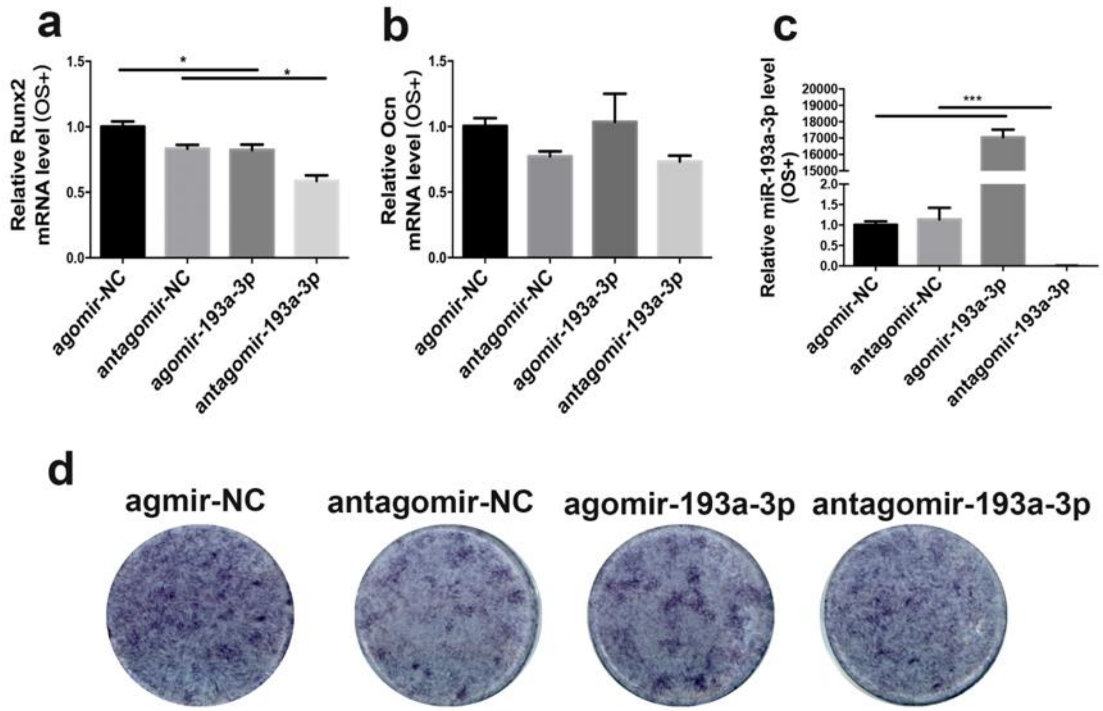

Supplementary Fig. 6. The promotion of osteogenic differentiation of hBMSCs by low hsa-miR-193a-3p expression was blocked by osteogenic differentiation medium (OS). Effect of agomir-193a-3p, antagomir, or their corresponding scrambled controls on the *Runx2* (**a**) and *Ocn* (**b**) mRNA levels in hBMSCs cultured in osteogenic differentiation medium. (**c**) The expression of miR-193a-3p at 48 hours post-transfection in osteogenic medium. Gross images (**d**) of ALP staining of hBMSCs treated with agomir-193a-3p, antagomir, or their corresponding scrambled controls for 5 days in osteogenic differentiation medium. Results are means ± SEM (n = 3). Samples were subjected to one-way ANOVA with Tukey *post hoc* test. Significant differences are denoted by *$p < 0.05$.

Supplementary Table 1 Flat（OS+）group on KEGG pathway enrichment

Rank	KEGG PATHWAY	*p* value
1	AGE-RAGE signaling pathway in diabetic complications	8.03395E-11
2	Amoebiasis	2.34792E-09
3	Pathways in cancer	2.47231E-09
4	Protein digestion and absorption	1.38926E-07
5	Focal adhesion	1.89465E-07
6	PI3K-Akt signaling pathway	2.9292E-07
7	cGMP-PKG signaling pathway	2.77904E-06
8	Hippo signaling pathway	3.60329E-06
9	Rap1 signaling pathway	4.79686E-06
10	@@Wnt signaling pathway	5.8927E-06
11	TNF signaling pathway	6.36061E-06
12	Small cell lung cancer	1.26062E-05
13	Proteoglycans in cancer	3.62616E-05
14	ECM-receptor interaction	4.00082E-05
15	Melanogenesis	4.87509E-05
16	Renin secretion	0.000144529
17	HTLV-I infection	0.00018663
18	Hypertrophic cardiomyopathy (HCM)	0.000198927
19	Calcium signaling pathway	0.000253525
20	Cytokine-cytokine receptor interaction	0.000268305

21	Platelet activation	0.000272684
22	Basal cell carcinoma	0.000273639
23	Signaling pathways regulating pluripotency of stem cells	0.000293983
24	Prolactin signaling pathway	0.000321215
25	FoxO signaling pathway	0.00056033
26	Colorectal cancer	0.00056603
27	Jak-STAT signaling pathway	0.000751165
28	Vascular smooth muscle contraction	0.000833465
29	@@TGF-beta signaling pathway	0.000891458
30	Phospholipase D signaling pathway	0.001010707
31	Chagas disease (American trypanosomiasis)	0.001014956
32	Prostate cancer	0.001296148
33	Dilated cardiomyopathy	0.001296148
34	Arrhythmogenic right ventricular cardiomyopathy (ARVC)	0.00161441
35	Long-term depression	0.002105404
36	Oxytocin signaling pathway	0.0022199
37	Inflammatory mediator regulation of TRP channels	0.002389074
38	Insulin signaling pathway	0.002481464
39	Cell adhesion molecules (CAMs)	0.00304302
40	Glucagon signaling pathway	0.003064878
41	Hepatitis B	0.003527827
42	Glycosaminoglycan biosynthesis - chondroitin sulfate / dermatan sulfate	0.003573867
43	MicroRNAs in cancer	0.003815343
44	Alanine, aspartate and glutamate metabolism	0.004166299
45	p53 signaling pathway	0.004305864
46	Endometrial cancer	0.004545765
47	@@Axon guidance	0.004807961
48	cAMP signaling pathway	0.005000776
49	Chronic myeloid leukemia	0.005710115

50	Ras signaling pathway	0.005867679
51	Glutamatergic synapse	0.006025044
52	Adherens junction	0.006109194
53	Glycosaminoglycan biosynthesis - heparan sulfate / heparin	0.006327502
54	Circadian entrainment	0.006613366
55	Acute myeloid leukemia	0.006821143
56	Thyroid hormone signaling pathway	0.007390009
57	Phosphatidylinositol signaling system	0.007826669
58	Neurotrophin signaling pathway	0.0081553
59	Estrogen signaling pathway	0.00826607
60	Regulation of actin cytoskeleton	0.008699829
61	EGFR tyrosine kinase inhibitor resistance	0.009509377
62	AMPK signaling pathway	0.009865073
63	Folate biosynthesis	0.009906553
64	HIF-1 signaling pathway	0.010210427
65	Adrenergic signaling in cardiomyocytes	0.010520728
66	Longevity regulating pathway - multiple species	0.011256282
67	Glioma	0.012024199
68	Long-term potentiation	0.012828241
69	mTOR signaling pathway	0.012892067
70	Central carbon metabolism in cancer	0.013669221
71	Type II diabetes mellitus	0.013679424
72	Mucin type O-Glycan biosynthesis	0.013964859
73	Gap junction	0.014120424
74	Rheumatoid arthritis	0.014120424
75	Viral carcinogenesis	0.014745751
76	Malaria	0.014751431
77	Cholinergic synapse	0.015087782
78	Measles	0.015958247

79	Fc gamma R-mediated phagocytosis	0.017376862
80	Inositol phosphate metabolism	0.017418218
81	Tight junction	0.018743836
82	Longevity regulating pathway	0.019189097
83	Toxoplasmosis	0.019719788
84	Glycerophospholipid metabolism	0.020143064
85	Leukocyte transendothelial migration	0.020580209
86	Gastric acid secretion	0.020655115
87	Platinum drug resistance	0.021818789
88	Endocrine resistance	0.022149211
89	Non-small cell lung cancer	0.023848057
90	Neuroactive ligand-receptor interaction	0.025130651
91	Bacterial invasion of epithelial cells	0.02557288
92	Arginine biosynthesis	0.025674019
93	Cell cycle	0.026308431
94	Retrograde endocannabinoid signaling	0.026567737
95	Glycerolipid metabolism	0.028650095
96	Osteoclast differentiation	0.034291654
97	Insulin resistance	0.037125789
98	Herpes simplex infection	0.037347936
99	ABC transporters	0.03959027
100	Pancreatic cancer	0.042099115
101	@@Sphingolipid metabolism	0.047793707
102	NF-kappa B signaling pathway	0.048673565
103	Hippo signaling pathway -multiple species	0.049718469

Supplementary Table 2 Random group on KEGG pathway enrichment

Rank	KEGG PATHWAY	*p* value
1	Protein digestion and absorption	1.79358E-09
2	ECM-receptor interaction	3.47032E-05
3	Focal adhesion	0.000192531
4	Pathways in cancer	0.000218548
5	MAPK signaling pathway	0.000358614
6	Hippo signaling pathway	0.000429406
7	Arrhythmogenic right ventricular cardiomyopathy (ARVC)	0.000835105
8	PI3K-Akt signaling pathway	0.001324168

9	Glycerolipid metabolism	0.001889905
10	Rap1 signaling pathway	0.003594875
11	AGE-RAGE signaling pathway in diabetic complications	0.003689107
12	Melanoma	0.004006342
13	HTLV-I infection	0.004116399
14	Glutathione metabolism	0.00740639
15	MicroRNAs in cancer	0.007735294
16	Hematopoietic cell lineage	0.008539659
17	Basal cell carcinoma	0.008888057
18	@@Pentose phosphate pathway	0.009568414
19	NF-kappa B signaling pathway	0.011075592
20	Arachidonic acid metabolism	0.013062871
21	Cytokine-cytokine receptor interaction	0.01417285
22	p53 signaling pathway	0.018314119
23	TNF signaling pathway	0.021641554
24	Glycosphingolipid biosynthesis - ganglio series	0.022460558
25	Bladder cancer	0.02264654
26	Complement and coagulation cascades	0.027817876
27	Platelet activation	0.031444199
28	Hypertrophic cardiomyopathy (HCM)	0.032305404
29	TGF-beta signaling pathway	0.033489632
30	Regulation of actin cytoskeleton	0.034055393
31	Ovarian steroidogenesis	0.036618953
32	Rheumatoid arthritis	0.038477357
33	Prostate cancer	0.03978713
34	Dilated cardiomyopathy	0.03978713
35	Osteoclast differentiation	0.040366132
36	FoxO signaling pathway	0.042536874
37	Terpenoid backbone biosynthesis	0.042803404

38	Transcriptional misregulation in cancer	0.043539533
39	Biosynthesis of unsaturated fatty acids	0.046111295
40	Renin-angiotensin system	0.046111295
41	Glycerophospholipid metabolism	0.048173965
42	Glycosaminoglycan biosynthesis - heparan sulfate / heparin	0.049509773

Materials and Methods

Fabrication of electrospun PLLA nanofiber. PLLA powder (0.7 g) was added to 10 mL of trifluoroethanol and stirred overnight. The solution was ejected from a 20 mL syringe with a steel needle (inner diameter: 0.5 mm), using a programmable syringe pump, into a conventional electrospinning apparatus (Elite; Ucalery, Beijing, China) at a rate of 0.7 mL/h. High-voltage equipment was used to provide a constant voltage (16 kV) to the tip of the needle when the fluid was ejected, and a metal plate (20 × 30 cm^2) was used as a collector at a distance of 15 cm from the tip of the needle, to obtain randomly arranged PLLA nanofibers (random group). To fabricate flat PLLA films (flat group), the PLLA polymer solution was cast on a flat glass plate at a thickness of 75 μm, and dried at 40°C for 6 h. The samples (PLLA nanofibers and flat films) were kept in a vacuum oven (DZF-6210; Blue Pard, Shanghai, China) at room temperature for 2 weeks to remove residual solvent. The nanotopography of the films was imaged by scanning electron microscopy (SU-8010; Hitachi, Tokyo, Japan), at an accelerating voltage of 15 kV.

Gel-MA hydrogel fabrication and rheological characterization. Gel-MA, an alterable elastic hydrogel, is commonly used as a mechanical model for cell culture. Briefly, Gel powder was added to phosphate-buffered saline (PBS) at 50°C and stirred until fully dissolved at 10% (w/v). Methacrylic anhydride was added to this solution at a rate of 0.3 mL/minute while stirring at 50°C until the target volume was reached. The mixture was next dialyzed against distilled water at 40°C for 2 weeks using 7000 molecular-weight (MW) cutoff dialysis tubing. The water was changed every day to remove salts and methacrylic acid. Subsequently, the Gel-MA solution was lyophilized and stored at −80°C for future use. Lyophilized Gel-MA at 3%, 5%, 7%, 10% and 20% (w/v) was dissolved in human mesenchymal stem cell basal medium containing 0.5% (w/v) 2-hydroxy-4′-(2-hydroxyethoxy)-2-methylpropiophenone (Aladdin, Shanghai, China) at 50℃ to prepare a pre-polymer solution. Two milliliters of the mixture were added to a 35-mm-diameter dish and exposed to ultraviolet (UV) light for 10 min. The rheological properties of the Gel-MA hydrogel were determined using a HAAKE RheoWin MARS 40 (Thermo Fisher, Waltham, MA, USA). Gel-MA hydrogels were placed within 35-mm-diameter parallel plates separated by 1.00 mm. Ampl sweeps were conducted from τ_0 0.01000–1,000 Pa (f = 1.000 Hz, T = 37℃). Frequency sweeps were performed at a constant strain from τ_0 0.03000–4 Pa according to the Gel-MA hydrogel concentration and frequencies of 0.1–14.00 Hz at 37℃.

Cell culture and seeding on PLLA nanofilms, Gel-MA, or six-well plates. The hBMSCs used in this research were supplied by Cyagen Biosciences Inc. (Guangzhou, China); they were obtained as surgical waste material from normal male donors aged 20–30 years. The culture medium was human mesenchymal stem cell-basal medium containing 5% (w/v) mesenchymal stem cell-qualified fetal bovine serum (FBS), 10 μg/mL (w/v) L-glutamine, and 100 IU/mL penicillin-streptomycin (Cyagen Biosciences Inc.). The medium was changed every 2–3 days. At 80–90% confluence, hBMSCs were detached with

0.25% (w/v) trypsin/ ethylenediaminetetraacetic acid (EDTA) (Gibco, Grand Island, NY, USA) and subcultured at a density of 5 × 10^5 cells per T75 flask, or plated onto six-well plates at a density of 1 × 10^5 cells per well. Fourth-passage hBMSCs were used in this study. PLLA nanofiber scaffolds and flat polymer films were cut into 4 × 4 cm sections and attached to the cap of centrifuge tubes (50 mL), as in our previous study. After placement within into six-well plates, these materials were sterilized with radiation for 12 h, and washed three times with PBS. Cells cultured on flat polymer films in OS comprising 50 mg/mL ascorbic acid, 10 mM sodium β-glycerol phosphate, and 10^{-8} M dexamethasone were used as flat (OS+) and random (OS+) positive controls. Negative controls were cultured on flat polymer films in normal culture medium without osteoinductive supplements.

Cell morphology and cytoskeletal remodeling. Morphological changes and cytoskeletal remodeling of hBMSCs were imaged by confocal microscopy. hBMSCs cultured on nanofibers for 4 h, and hBMSCs cultured on Gel-MA for 24 h, were washed with PBS, fixed in 4% (w/v) paraformaldehyde for 15 min, washed twice in PBS, permeabilized with 0.2% (w/v) Triton X-100 for 10 min, and blocked with 5% (w/v) bovine serum albumin (BSA) for 60 min at room temperature. The flat or random PLLA membranes were stained with tetramethylrhodamine isothiocyanate (TRITC) phalloidin (100 nM; Solarbio, Shanghai, China) 40 min at room temperature, followed by counterstaining with 1 μg/mL 4',6-diamidino-2-phenylindole (DAPI) (blue) for 10 min. The cells were visualized by confocal laser scanning microscopy (TCS SP8; Leica, Wetzlar, Germany). Image Pro Plus software (Media Cybernetics, Silver Spring, MD, USA) was used to evaluate the effect of Gel-MA concentration on cell morphology. The shape of the cell and nucleus of at least 30 cells per group were analyzed.

Quantitative reverse transcriptase-polymerase chain reaction. The PLLA nanofilms or Gel-MA loaded with cells for in vitro culture were prepared as described previously. Total RNA of cells cultured on PLLA nanofilms was extracted using TRIzol reagent (Invitrogen, Carlsbad, CA, USA). Total RNA of the cells loaded on Gel-MA was extracted using the RNAprep Pure Cell Kit (DP430; Tiangen, Beijing, China). mRNA was reverse-transcribed into cDNA using the PrimeScript® RT Reagent Kit (TaKaRa, Tokyo, Japan) at 37°C for 15 min and 85°C for 5 s according to the manufacturer's protocol. The synthesized cDNA samples were subjected to qRT-PCR to determine the expression levels of *Runx2, Ocn*, *Map3k3*, *Bsp*, *Erk1*, *Jnk*, and *Erk5* using SYBR Green Master Mix (Roche Diagnostics Ltd., Mannheim, Germany) and a 7500 ABI Real-Time PCR System (Applied Biosystems, Foster City, CA, USA). Relative gene expression levels were calculated using the $2^{(-\Delta\Delta CT)}$ method. Briefly, the mRNA and miR-193a-3p data were normalized to the expression of glyceraldehyde-3-phosphate dehydrogenase (GAPDH) and U6 (U6 spliceosomal RNA), respectively. The following primer sequences were used for qRT-PCR: *Gapdh* (5'-CCTCTGACTTCAACAGCGAC-3', 5'-TCCTCTTGTGCTCTTGCTGG-3'), *Runx2* (5'-TCACCTCAGGCATGTCCCTCGGTAT-3', 5'-TGGCTTCCATCAGCGTCAACACC-3'), *Ocn* (5'-CACTCCTCGCCCTATTGGC-3', 5'-CCCTCCTGCTTGGACACAAAG-3'), *Map3k3* (5'-GGCGAATTATAGCGTTCAGCC-3', 5'-GGGACAACAGCAATATCCTAAGG-3') *Bsp* (5'-GGAGACTTCAAATGAAGGAG-3', 5'-CAGAAAGTGTGGTATTCTCAG-3'), *Erk1* (5'-ACTCCAAAGCCCTTGACCTG-3', 5'-GACTGGCCCACCTCATCC-3') *Jnk* (5'-CCACCACCAAAGATCCCTGA-3', 5'-GCTGCACCTAAAGGAGAGGG-3'), *Erk5* (5'-GCAGGTGGCCATCAAGAAGA-3', 5'-TCCAGGACCACGTAGACAGA-3'), *U6* (5'-CGCTTCGGCAGCACATATAC-3', 5'-TTCACGAATTTGCGTGTCATC-3'), and hsa-miR-193a-3p (5'-ATGCTCAAACTGGCCTACAAAG-3', 5'-TATGGTTGTTCTGCTCTCTGTCTC-3').

miRNA array, gene array and bioinformatics analysis. Total RNA, including small RNAs, from hBMSCs cultured for 14 days on PLLA nanofibers (random) and flat polymer films with (flat OS+) or without OS (flat), was extracted using TRIzol reagent (Invitrogen) and purified using the mirVana miRNA Isolation Kit (Ambion, Austin, TX, USA) according to the manufacturer's protocol. miRNA profiling was performed using an Agilent miRNA array (CapitalBio Corp, Beijing, China). The Agilent array has eight identical arrays per slide (8 × 60 K format), with each array containing probes interrogating 2,549 human mature miRNAs from miRBase R21.0 (www.mirbase.org). Each miRNA was detected by probes repeated 30 times. The array also contained 2,164 Agilent control probes. The miRNA profiling was performed using the Agilent miRNA array. The Agilent array was designed with eight identical arrays per slide (8 × 60 K format), with each array containing probes interrogating 2,549 human mature miRNAs from miRBase R21.0. Each miRNA was detected by probes repeated 30 times. The array also contained 2,164 Agilent control probes.

Briefly, 200 ng of total RNA were used to synthesize first-strand cDNA followed by double-stranded cDNA using a Message Amp Premier RNA Amplification Kit and PCR apparatus (MJ, PTC-225; Ambion). Biotin-labeled cRNA was synthesized using a MessageAmp Premier RNA Amplification Kit (Ambion). The concentration of cRNA was measured using a NanoDrop ND-1000 instrument, and 15 μg of fragmented cRNA was hybridized to each GeneChip Human Genome U133 Plus 2.0 Array (Affymetrix, Santa Clara, CA, USA), which contains more than 54,000 probe sets to cover over 47,000 transcripts and variants, at 45°C for 16 h (Affymetrix Hybridization Oven 640) according to the manufacturer's instructions. After hybridization, the arrays were washed, stained with streptavidin phycoerythrinonan using an Affymetrix Fluidics Station 450, and scanned using an Affymetrix Scanner 3000 7G. Three replicates of the microarray experiment were performed until high reproducibility was achieved. Each replicate comprised pooled cell lysates from three wells.

Bioinformatics analysis. The miRNA array data were , normalized, summarized and subjected to quality control using GeneSpring software v. 12 (Agilent). The default 90th percentile normalization method was applied for data pre-processing. To identify differentially expressed genes, we used threshold values of $\geq$ 2.5 and ≤ -2.5-fold changes, and a *t*-test p-value of 0.05. The raw data were log2-transformed and median-centered by genes using the Adjust Data function of CLUSTER 3.0 software (Stanford University School of Medicine, Stanford, CA, USA), and further analyzed by hierarchical clustering and heatmaps with average linkage. We performed tree visualization in using Java TreeView (Stanford University School of Medicine). In the heatmap, red denotes high relative expression, and green low relative expression. A PCA was performed to determine the gene expression trends of the samples.

Ontology and pathway analysis. The number of genes potentially regulated by one miRNA ranges from several hundreds to a couple of thousand. The predicted mRNA targets of downregulated miRNAs were picked up by no less than 7 out of 12 commonly used predicting databases: miRWalk, microT v4, miRanda, mirbridge, miRDB, miRMap, miRNAMap, Pictar2, PITA, RNA22, RNAhybrid, and Targetscan. The predicted mRNA targets that overlapped with upregulated mRNAs in gene chips subjected to ontology analysis with KOBAS 3.0 software were identified to determine the enriched biological pathways regulated by the candidate downregulated miRNAs.

miRNA transfection. hBMSCs were cultured to 70% confluence in six-well plates in basal medium with 5% (v/v) FBS. Next, 50 nM agomir or 100 nM antagomir (GenePharma) was transfected into cells using

lipofectamine RNAi-max (Invitrogen) to potentiate or block the activity of miR-193a-3p, respectively. Negative controls (double-stranded agomir and single-stranded antagomir validated by the manufacturer to have no homology with any published human or mouse miRNA) were also performed for both reactions. At 8 h after transfection, the transfection medium was replaced with fresh complete medium. At 48 h after transfection, RNA was extracted, and qRT-PCR analysis was performed. At 72 h after transfection, proteins were extracted, and a Western blotting analysis was performed. For alkaline phosphatase (ALP) staining, the cells were subjected to transfection for 5 days.

Western blotting. Cells were lysed in radioimmunoprecipitation assay buffer (RIPA) lysis buffer (P0013B; Beyotime, Jiangsu, China) with protease and phosphatase inhibitors (Halt; Thermo Scientific) on ice for 30 min. Protein fractions were collected by centrifugation at 12,000 g at 4°C for 30 min. The protein concentrations were determined using a BCA Protein Assay Kit (P0012; Beyotime), and whole lysates were mixed with 6× sodium dodecyl sulfate (SDS) loading buffer (P0015F; Beyotime) at a 1:5 ratio. The samples were heated for 5 min at 100°C and, subjected to electrophoresis on SDS-polyacrylamide gels; the resolved proteins were transferred to polyvinylidene difluoride (PVDF) membranes. The membranes were blocked with 5% (w/v) BSA and incubated with specific antibodies overnight. The following antibodies were used: anti-GAPDH (1:2,500; ab9485; Abcam), anti-RUNX2 (1:1,000; 12556; Cell Signaling Technology), anti-OCN (1:500; DF12303; Affinity), anti-MAP3K3 (1:1,000; ab40756; Abcam), anti-phospho-Erk1/2 (Thr202/Tyr204) (1:1000; 9101; Cell Signaling Technology), anti-Erk1/2 (1:1,000; 9102; Cell Signaling Technology), anti-phospho-SAPK/JNK (Thr183/Tyr185) (1:500; 9255, Cell Signaling Technology), anti-SAPK/JNK (1:1,000; 9252; Cell Signaling Technology), anti-phospho-ERK5 (Thr218/Tyr220) (1:500; 3371; Cell Signaling Technology), and anti-ERK5 (1:1,000; 3372; Cell Signaling Technology). The target proteins were probed with a horseradish peroxidase (HRP)-conjugated goat anti-rabbit IgG (A0208; Beyotime) or HRP-conjugated goat anti-mouse IgG (A0216; Beyotime), and autoradiograms were performed using the cECL Western Blot Kit (CW0049; CWBio, Beijing, China).

Alkaline phosphatase staining. ALP in cell layers was detected as follows. Cultured cells were rinsed with PBS three times and fixed with 4% (w/v) paraformaldehyde for 15 min at room temperature. ALP staining was performed using the BCIP/NBT Alkaline Phosphatase Colour Development Kit (C3206; Beyotime) according to the manufacturer's instructions. The samples were placed in ALP substrate staining solution for 15 min and rinsed three times in deionized water. The entire procedure was protected from light.

Luciferase reporter assay. *Map3k3* mRNA 3'UTRs containing the miR-193a-3p–binding sequences for human *Map3k3* (NM_203351) were amplified from human genomic DNA by PCR. Double-stranded oligonucleotides corresponding to the WT MAP3K3 3'UTR or MUT MAP3K3 3'UTR miR-193a-3p binding site in the 3'UTR of *Map3k3* were synthesized and subcloned into the GV272 reporter vector (GV272: SV40-Luciferase-MCS-Poly A; GeneChem, Shanghai, China). The miR-193a-3p recognition elements were as follows: 5'-ACCGAGGGCTTGCAGTGCAAAGCCAGGCCAGTGTTGCGCATTA -3' (WT MAP3K3 3'UTR) and 5'-ACCGAGGGCTTGCAGTGCAAAGCCATTAACTGGTTGCGCATTA-3' (MUT MAP3K3 3'UTR). Cells were seeded into a 24-well plate and co-transfected with the appropriate plasmid and miR-193a-3p agomir, antagomir, or scrambled control using Lipofectamine™ 3000 (Invitrogen). Luciferase assays were performed using the Dual-Luciferase Reporter Assay System

(E1910; Promega, Madison, WI, USA) at 48 h after transfection. Normalized luciferase activity is presented as luciferase activity ÷ Renilla luciferase activity.

Lentivirus infection. Lentiviruses carrying shRNA targeting human *Map3k3* and lentiviral vectors (GV493) overexpressing human *Map3k3* (NM_203351) (GV358) were procured from GeneChem. The viruses were used to infect hBMSCs in the presence of Polybrene. Forty-eight hours later, the infected hBMSCs were cultured in medium containing puromycin for the selection of stable clones. The clones with stably knocked down or *Map3k3* overexpression was identified and validated by qPCR and western blotting. The shRNA sequences are as follows: MAP3K3 no. 1: 5'-ACCTCTTGATCTACATTACAT-3'; MAP3K3 no. 2, 5'-GTGCGAGATCCAGTTGCTAAA-3'; and the non-targeting control, 5'-TTCTCCGAACGTGTCACGT-3'.

Alizarin red staining. Cells transfected with lentivirus were seeded in six-well plates. After 21 days, the cells were fixed in 70% (v/v) ice-cold ethanol for 30 min and rinsed with double-distilled H_2O. The cells were stained with 40 mM Alizarin red S (pH 4.0; Sigma-Aldrich, St. Louis, MO, USA) for 15 min with gentle agitation. Finally, the cells were rinsed five times with double-distilled H_2O, and then for 15 min with 1× PBS with gentle agitation.

Animals and surgical procedures.

24 male Sprague-Dawley rats (6 weeks old) were purchased from the Beijing Vital River Laboratory Animal Technology Co., Ltd. (Beijing, China), which were divided into 4 groups at two time points (6 samples per group). All animal surgical procedures were approved by the Institutional Animal Care and Use Committee of Peking University Health Science Center. All procedures were performed under general anesthesia by intraperitoneal injection of pentobarbital (50 mg/kg body weight). The rats were randomly divided into the random, scrambled, miR-193a-3p agomir and miR-193a-3p antagomir groups. Surgical procedures were performed under sterile conditions. A 15–20 mm incision was made on the middle of the calvarium, and a 5-mm-diameter osteotomy defect was created at the lateral side of the cranial sagittal suture using a trephine bur with sterile saline irrigation. The sites were covered by a PLLA membrane, and lyophilized with H_2O, scrambled agomir, miR-193a-3p agomir, and miR-193a-3p antagomir. The surgical field was closed in the muscular layer, followed by the external skin layer, using 5-0 synthetic resorbable sutures.

Micro-computed tomography. At 4 and 8 weeks post-operation, calvaria samples were harvested, fixed in 4% (w/v) PBS formalin solution for 24 h, and stored in PBS until micro- micro-CT scans were performed. Files were reconstructed using a modified Feldkamp algorithm, which was created using microtomographic analysis software (Inveon; Siemens, Munich, Germany). After 3D visualization, the BV and BMD were determined in the region of interest (ROI).

Histological Analysis. Tissue processing and sectioning were carried out as previously described[22]. Briefly, tissue samples were fixed in 10% neutral buffered formalin for 7 d, decalcified and dehydrated according to standard protocols, embedded in paraffin and sectioned at 5 μm thickness. Hematoxylin and eosin (H&E) staining and Masson's staining were performed separately on tissue sections, according to the manufacturer's protocols, and images were captured under light microscope (CX21, Olympus, Japan).

Statistical analysis. For each data set, three independent experiments in triplicates were performed to confirm the replicability of the experimental data, but only the results of one representative experiment was presented. We utilized Shapiro-Wilks test to evaluate whether the data is normally distributed before parametric statistical analysis. Data are presented as means ± standard deviation (SD). Comparisons between two groups were carried out by two-tailed unpaired Student's *t*-test. For multiple-group comparisons (at different time points), one-way and two-way analyses of variance (ANOVA) were used to evaluate whether differences were significant. *Post hoc* analysis using Bonferroni correction was performed.
